# Edge Artifacts in Point Spread Function-based PET Reconstruction in Relation to Object Size and Reconstruction Parameters

**DOI:** 10.22038/aojnmb.2017.8802

**Published:** 2017

**Authors:** Yuji Tsutsui, Shinichi Awamoto, Kazuhiko Himuro, Yoshiyuki Umezu, Shingo Baba, Masayuki Sasaki

**Affiliations:** 1 Division of Radiology, Department of Medical Technology, Kyushu University Hospital, Fukuoka, Japan; 2 Department of Clinical Radiology, Kyushu University Hospital, Fukuoka, Japan; 3 Department of Health Sciences, Faculty of Medical Sciences, Kyushu University, Fukuoka, Japan

**Keywords:** Edge artifact, PET, Point-spread function

## Abstract

**Objective(s):**

We evaluated edge artifacts in relation to phantom diameter and reconstruction parameters in point spread function (PSF)-based positron emission tomography (PET) image reconstruction.

**Methods:**

PET data were acquired from an original cone-shaped phantom filled with ^18^F solution (21.9 kBq/mL) for 10 min using a Biograph mCT scanner. The images were reconstructed using the baseline ordered subsets expectation maximization (OSEM) algorithm and the OSEM with PSF correction model. The reconstruction parameters included a pixel size of 1.0, 2.0, or 3.0 mm, 1-12 iterations, 24 subsets, and a full width at half maximum (FWHM) of the post-filter Gaussian filter of 1.0, 2.0, or 3.0 mm. We compared both the maximum recovery coefficient (RC_max_) and the mean recovery coefficient (RC_mean_) in the phantom at different diameters.

**Results:**

The OSEM images had no edge artifacts, but the OSEM with PSF images had a dense edge delineating the hot phantom at diameters 10 mm or more and a dense spot at the center at diameters of 8 mm or less. The dense edge was clearly observed on images with a small pixel size, a Gaussian filter with a small FWHM, and a high number of iterations. At a phantom diameter of 6-7 mm, the RC_max_ for the OSEM and OSEM with PSF images was 60% and 140%, respectively (pixel size: 1.0 mm; FWHM of the Gaussian filter: 2.0 mm; iterations: 2). The RC_mean_ of the OSEM with PSF images did not exceed 100%.

**Conclusion:**

PSF-based image reconstruction resulted in edge artifacts, the degree of which depends on the pixel size, number of iterations, FWHM of the Gaussian filter, and object size.

## Introduction

^18^F-fluoro-2-deoxy-2D-glucose (^18^F-FDG) positron emission tomography/computed tomography (PET/CT) is employed to manage a wide range of malignant tumors (1-3). Although an iterative reconstruction algorithm improves the quality of a reconstructed PET image (4), the partial volume effect due to low spatial resolution still affects the quantitative accuracy of the PET/CT images. To resolve this problem, point spread functions (PSFs) in the field of view (FOV) were recently incorporated into the reconstruction algorithm. The use of the iterative reconstruction method in combination with PSF modeling has been shown to improve spatial resolution and contrast of the image (5-9).

However, PSF-based reconstruction is known to cause edge artifacts (also known as Gibbs artifacts), which appear as an overshoot at the sharp transition of intensity of the phantom (10). Furthermore, edge artifacts at both sides of the transition are thought to merge and result in a high degree of overestimation of radioactivity in small regions (11-13), which could cause a challenge in the quantitation and standardization of PET imaging.

Bai et al. reported that overestimation due to edge artifacts depends on both the cylinder size and the radioactivity ratio (12). We previously reported the relationship between edge artifacts and radioactivity ratios (14). We used an NEMA IEC Body Phantom consisting of six spheres of 37, 28, 22, 17, 13, and 10 mm in diameter. Edge artifacts were prominent in the 13-mm-diameter sphere with high sphere-to-background ratio (SBR) and resulted in overestimation of radioactivity. Influence of iteration number on the appearance of edge artifact was also examined. However, we discretely evaluated only a limited number of predetermined sphere sizes. The relationship between edge artifacts and the precise phantom diameter must be examined. Furthermore, influence of other reconstruction parameters, such as the pixel size and the size of post-reconstruction filter, are considered to influence the appearance of edge artifact.

The aim of this study was to evaluate the edge artifacts on PET images that were reconstructed using the PSF algorithm and their relationship with object size and reconstruction parameters.

## Methods

### Phantom

**Figure 1:**
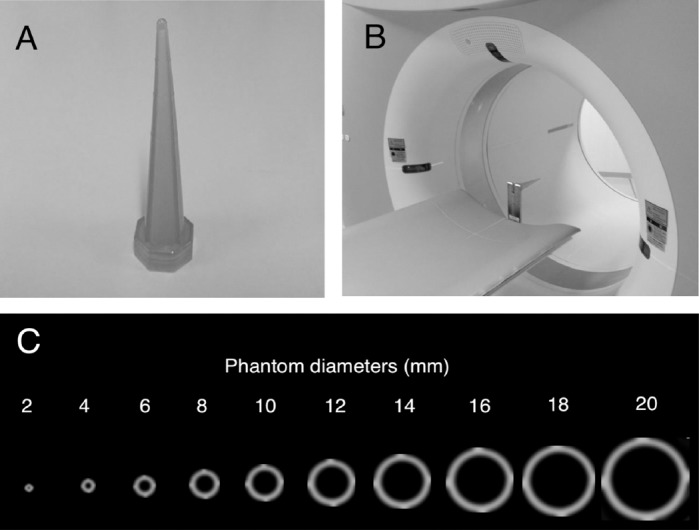
Cone-shaped phantom: (A) exterior, (B) positioning on PET/CT scanner, and (C) CT images

The phantom used in the present study was a 115 mm long cone-shaped plastic bottle, with its largest diameter at 20 mm (Figure 1). The phantom was filled with ^18^F solution (21.9 kBq/mL).

### PET/CT Scanner

The PET/CT data was acquired using a Biograph mCT (Siemens Healthcare, Erlangen, Germany). This PET scanner has three rings containing 144 lutetium orthosilicate detectors and each block is 4×4×20 mm. The axial FOV was 16.2 cm and the transaxial FOV was 70 cm. The coincidence time window was 4.1 ns, and the spatial resolution at 1 and 10 cm was 4.4 and 4.9 mm FWHM, respectively. The system sensitivity when the line source was 0 and 10 cm from the center of the FOV was 0.96% and 0.94%, respectively.

### Data Acquisition and Image Reconstruction

The phantom was positioned at the center of the PET FOV with a background of air. The emission data was acquired in the list mode for 10 min. The PET image was reconstructed using 3D ordered subsets expectation maximization (OSEM) with and without a PSF algorithm with correction of CT attenuation. Time-of-flight information was not used for reconstruction. The reconstruction parameters were as follows: pixel size: 1.0×1.0, 2.0×2.0, and 3.0×3.0 mm, FWHM of the post-filter of the Gaussian filter 1.0, 2.0, and 3.0 mm, iterations: 1, 2, 4, 6, 8, 10, and 12, subsets: 24, and slice thickness: 3.0 mm.

The CT scanning parameters included: 120 kV, 100 mAs (Eff.mAs), 512×512 matrix, 32 slices, 3.0 mm slice thickness, and 500 mm transaxial FOV.

### Data analysis

We measured both the maximum and mean counts of the phantom at different diameters. The circular regions-of-interest (ROIs) with diameters equal to those of the phantom were placed using a CT image as a reference.

The maximum and mean counts were analyzed using the recovery coefficient (RC), which was calculated using the following equations:


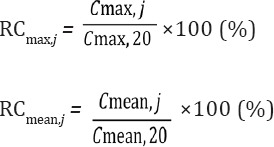


where *j* is the phantom diameter (mm), *C*_max,20_ is the maximum count of a 20-mm diameter phantom, *C*_max,*,j*_ is the maximum count of a *j* diameter phantom, *C*_mean,20_ is the mean count in the ROI of a 20-mm diameter phantom, and *C*_mean,*,j*_ is the mean count in the ROI of a *j* diameter phantom.

## Results

### The pixel size

**Figure 2:**
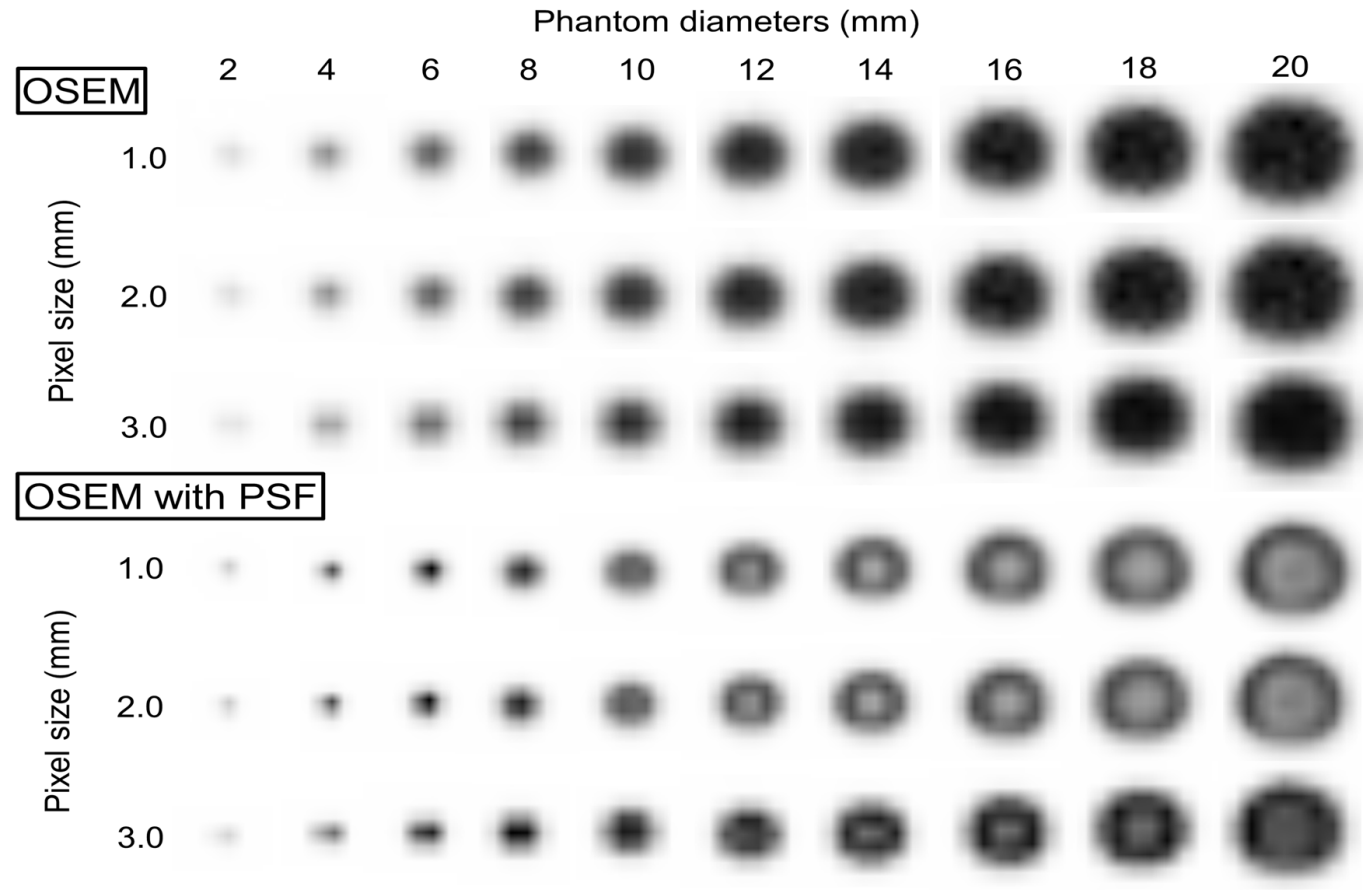
Reconstructed PET images at different phantom diameters using OSEM and OSEM with PSF (iteration 2, subset 24). The pixel size ranged from 1.0 to 3.0 mm. The OSEM images do not show any edge artifacts. However, in the OSEM with PSF images, a dense edge delineates the phantom at larger diameters and a sharp peak is observed at the center of the phantom at smaller diameters

Figure 2 displays PET images of the phantom at different diameters and three different pixel sizes (1.0×1.0, 2.0×2.0, and 3.0×3.0 mm). The images were reconstructed using two iterations, 24 subsets, and a 1 mm FWHM of Gaussian filter. The OSEM images had no edge artifacts, but the OSEM with PSF images had a dense edge delineating the hot phantom when the diameter was 10 mm or more and a dense spot at the center when the diameter was 8 mm or less. The dense edge was clearly visible on images with a small pixel size.

**Figure 3:**
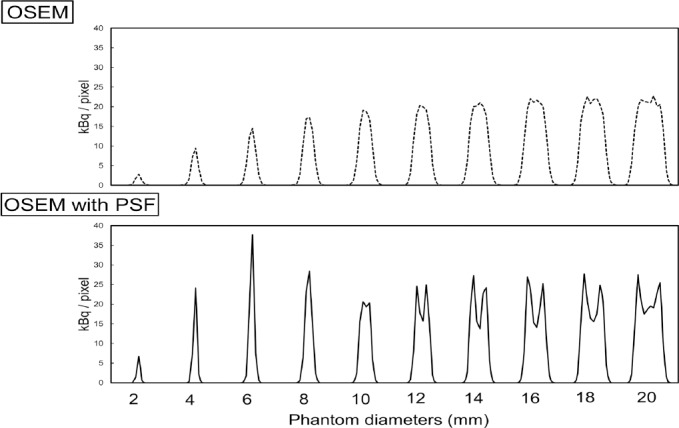
Profiles through the center of the phantom as the diameter ranged from 2 to 20 mm and the pixel size was 1.0 mm. Top panel: OSEM, bottom panel: OSEM with PSF.

**Figure 4:**
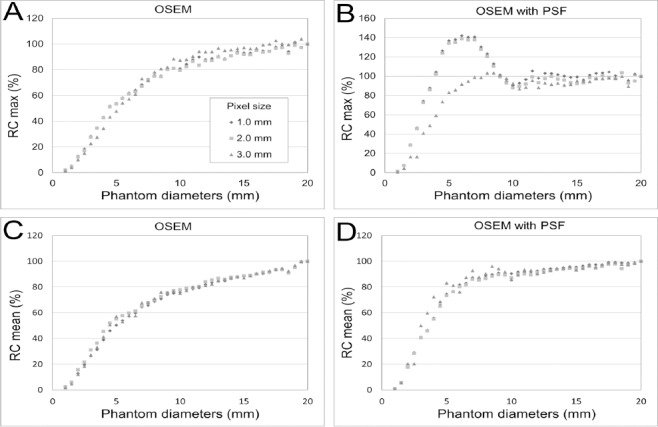
Relationship between phantom diameter and recovery at different pixel sizes; (A) RC_max_ of the OSEM image, (B) RC_max_ of the OSEM with PSF image, (C) RC_mean_ of the OSEM image, and (D) RC_mean_ of the OSEM with PSF image

Figure 3 shows the profiles through the center of the phantom at diameters of 2-20 mm. Figure 4 presents RC_max_ and RC_mean_ as functions of the phantom diameter for different pixel sizes. For the OSEM images, the RC_max_ and RC_mean_ gradually decreased as the diameter of the phantom decreased. The curves for the three pixel sizes do not differ. The RC_max_ and RC_mean_ for the OSEM with PSF images were higher than those for the OSEM images. Although the RC_max_ was higher than the RC_mean_ for both OSEM and OSEM with PSF images, the RC_max_ was overestimated for OSEM with PSF images at the transition area of intensity of the phantom. On images with a pixel size of 1.0 and 2.0 mm, the dense edge was narrow and the degree of overestimation was high compared to the images with a pixel size of 3.0 mm. As shown in Figure 4(B), the RC_max_ was overestimated for the 1.0 and 2.0 mm pixel images at phantom diameters of 6-7 mm and for the 3.0 mm pixel images at phantom diameters of 8-9 mm. The RC_mean_ was not overestimated for the OSEM with PSF images.

### Iterations

**Figure 5:**
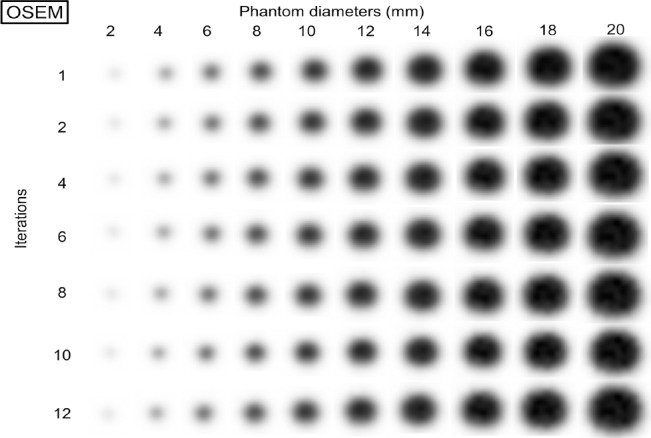
PET images of the phantom at different diameters reconstructed by OSEM using 1-12 different iterations; no images show edge artifacts

**Figure 6:**
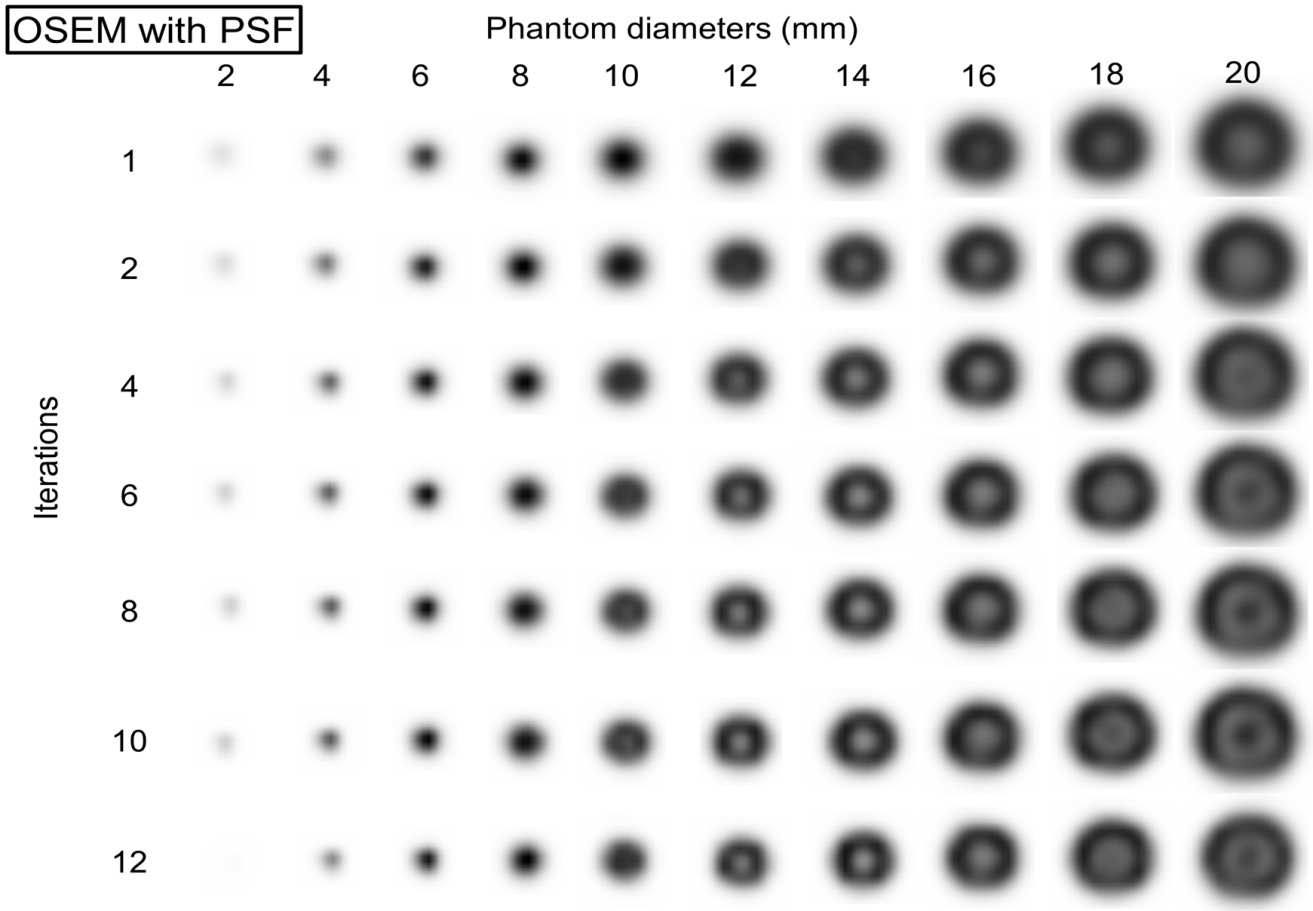
PET images of the phantom at different diameters reconstructed by OSEM with PSF using 1-12 iterations; a dense edge delineates the phantom at larger diameters and a sharp peak is observed at the center of the phantom at smaller diameters. As the number of iterations increases, the dense edge narrows and the overshoot at the center is visible at larger diameters

Figures 5 and 6 show the OSEM and OSEM with PSF PET images, respectively, for different phantom diameters and iterations (1, 2, 4, 6, 8, 10, and 12). The image reconstruction parameters were 1.0×1.0 mm pixel size, 24 subsets, and 2.0 mm FWHM of the Gaussian filter. The OSEM images (Figure 5) show no edge artifacts, but in OSEM with PSF images (Figure 6), there is an overshoot of the sharp transition of intensity at different diameters and numbers of iterations. As the number of iterations increased, the dense edge became narrower and the undershoot, which was just inside the dense edge, became more visible.

**Figure 7:**
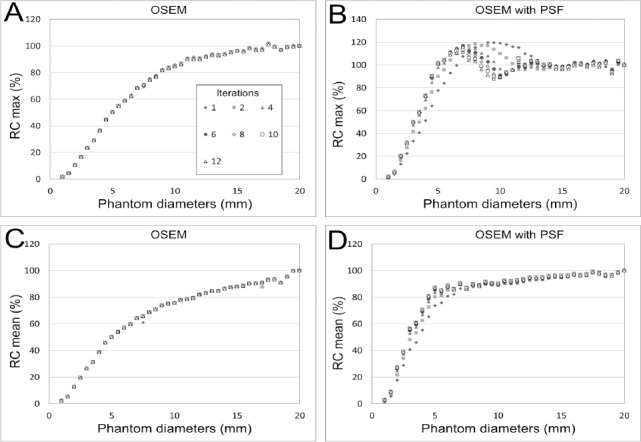
Relationship between the phantom diameter and recovery for different numbers of iterations; (A) RC_max_ of the OSEM image, (B) RC_max_ of the OSEM with PSF image, (C) RC_mean_ of the OSEM image, and (D) RC_mean_ of the OSEM with PSF image

Furthermore, the second peak at the center of the phantom appeared. Figure 7 exhibits the RC_max_ and RC_mean_ as a function of different phantom diameters for different numbers of iterations. The number of iterations did not affect the degree of overestimation. As the number of iterations increased, the largest overestimations occurred at small diameters. The ringing frequency raised (the wavelength decreased) as the number of iterations increased. However, the RC_mean_ values of the OSEM with PSF images were not affected by the edge artifact.

### FWHM of Gaussian filter

**Figure 8:**
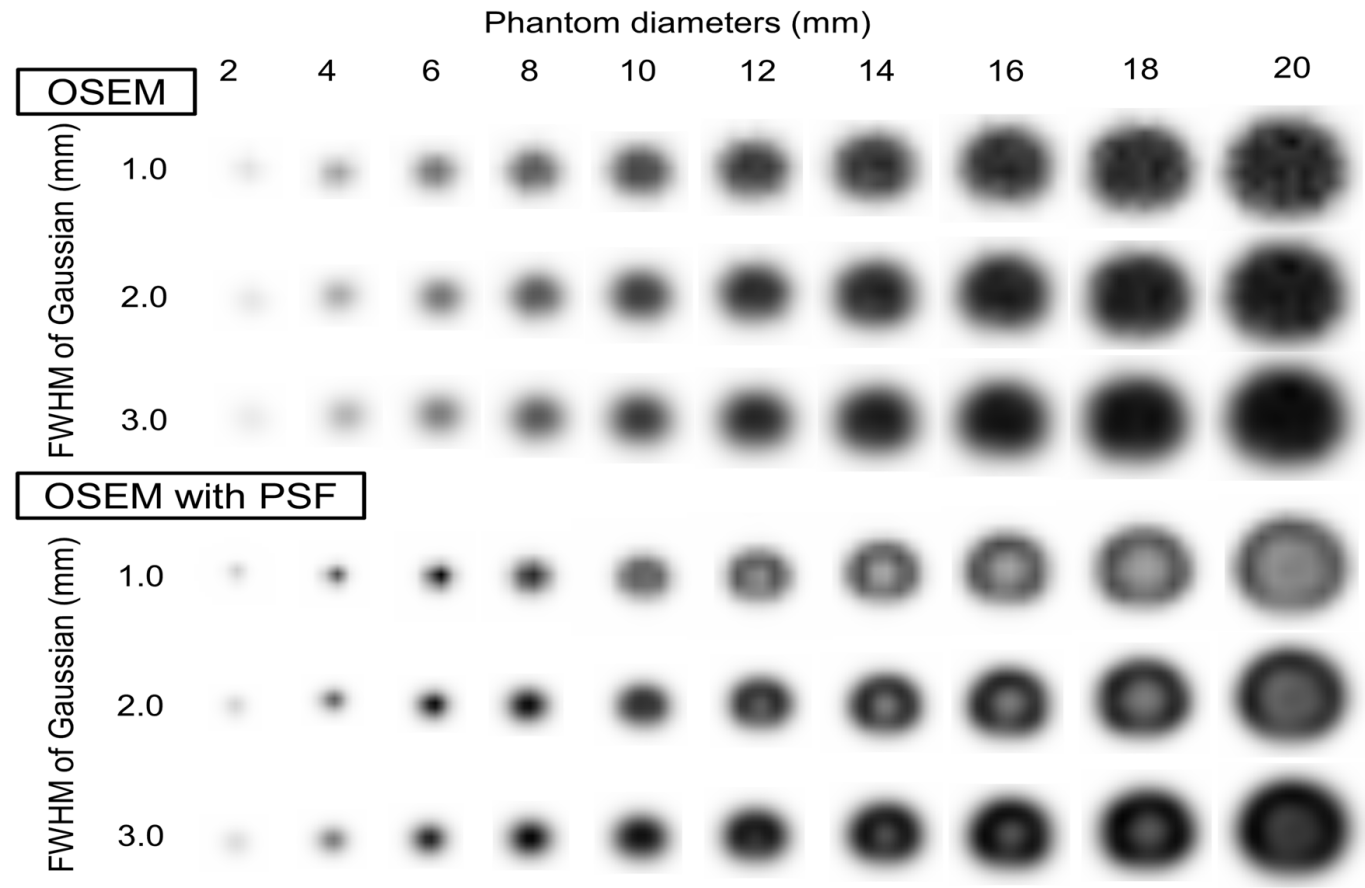
PET images captured with the FWHM of the Gaussian filter ranging from 1.0 to 3.0 mm. The OSEM images show no the edge artifacts, but in the OSEM with PSF images, the dense edge delineating the phantom becomes wider and blurry

Figure 8 presents the PET images of the phantom at different diameters and three different FWHM values of the Gaussian filter (1.0, 2.0, and 3.0 mm). The images were reconstructed using two iterations, 24 subsets, and a 1.0×1.0 pixel size. The OSEM images show no edge artifacts, whereas the edge artifacts in the OSEM with PSF images appear as overshoots of the sharp transition of intensity of the phantom. The edge artifact became wide and blurry as the FWHM of the Gaussian filter increased.

**Figure 9:**
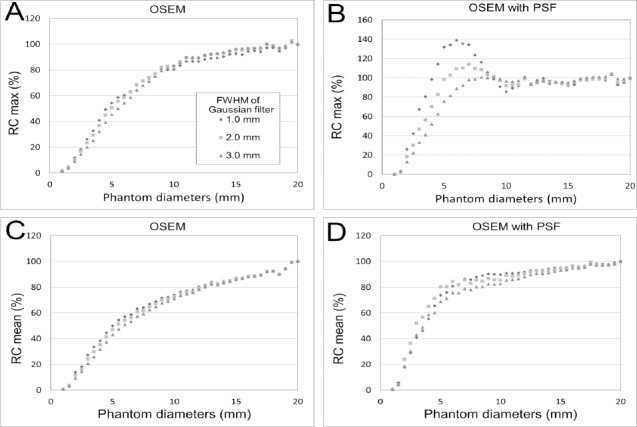
Relationship between the phantom diameter and recovery for Gaussian filters with different FWHM values; (A) RC_max_ of the OSEM image, (B) RC_max_ of the OSEM with PSF image, (C) RC_mean_ of the OSEM image, and (D) RC_mean_ of the OSEM with PSF image

Figure 9 shows the RC_max_ and RC_mean_ at different phantom diameters for three different FWHM values of the Gaussian filter. For the OSEM with PSF images, the degree of overestimation due to edge artifacts was prominent when the FWHM was small (Figure 9[B]). The RC_max_ of the images with a 1.0 mm FWHM was 40% higher when the diameter was 6 mm, while with a 2.0 mm FWHM it was 14% higher when the diameter was 7 mm, and with 3.0 mm FWHM it was 1% higher when the diameter was 8 mm than that of the image when the diameter was 20 mm (Figure 9[B]). However, the RC_mean_ of the OSEM image (Figure 9[C]) did not show any overestimation. The RC_max_ of the OSEM in the image with a 1.0 mm FWHM was 40% lower when the diameter was 6 mm. RC_max_ with a 2.0 mm FWHM was 31% lower when the diameter was 7 mm and with 3.0 mm FWHM it was 25% lower when the diameter was 8 mm than that of the image when the diameter was 20 mm (Figure 9[A]).

## Discussion

We examined the edge artifacts in PET images of a cone-shaped phantom that were reconstructed using the OSEM with PSF algorithm. The edge artifact appeared as an overshoot or ringing at the sharp transition of intensity of the phantom, and there was an overestimation of the RC_max_ when the diameter was 8 mm. This could result in an overestimation of up to 40% at the center of the phantom. The magnitude of the overshoot and the diameter of the observed overshoot are depended on the pixel size, number of iterations, and FWHM of Gaussian filter.

Our study also found a similar degree of overestimation at pixel sizes of 1.0×1.0 and 2.0×2.0 mm, but it was minimal at a pixel size of 3.0×3.0 mm. We also observed narrow and large overshootings at small pixel sizes. Bai et al. (12) observed a 50% overestimation with an 8-mm-diameter cylindrical phantom. They also examined edge artifacts from using a Biograph mCT 64 scanner with pixels of 1.06×1.06 mm.

Our results suggested that a small pixel size resulted in narrow and large artifacts. Although our previous study showed the maximum overestimation in a sphere with 13-mm diameter, the present study demonstrated it at 8-mm diameter of cone-shaped phantom. This difference may be resulted from the difference in pixel size. Furthermore, the different shape of phantoms may result in this difference because the edge artifact appeared 3 dimensionally in sphere phantoms.

In our study, an increase in the number of iterations resulted in narrow edge artifacts. Furthermore, low radioactivity just inside the dense edge and the second peak at the center of the phantom became apparent. Panin et al. (6) observed that the recovery of small spheres was improved when more iterations were performed in the PSF reconstruction. Bai et al. (12) reported that the RC continued to increase using OSEM with PSF, even after 12 iterations. Furthermore, Tong et al. (15) reported that an increase in the number of iterations led to a rise in the frequency and a decrease in the wavelength of the radioactivity profile. These results suggest that enhanced number of iterations in the PSF algorithm increase the visibility of the edge artifacts.

The smoothing filter for the PET image was introduced to equalize the standardized uptake values (SUVs) obtained using different scanners or reconstruction protocols (16, 17).

Panin et al. (6) addressed that post-smoothing with a 7 mm Gaussian filter reduced the different SUVs between the OSEM and OSEM with PSF images. Alessio et al. (7) stated that after applying a 5 mm Gaussian filter, the RC curve of the OSEM with PSF image was very similar to that of an OSEM image without smoothing. In our study, a 3.0 mm FWHM did not eliminate edge artifacts but did suppress overestimation. The difference in results obtained with a 5 mm FWHM Gaussian filter and a 3 mm FWHM Gaussian filter may arise from the use of different reconstruction parameters, especially pixel size.

A clinical study by Andersen et al. (18) showed that the mean relative changes in SUV_max_ and SUV_mean_ were 46±27% and 45±27%, respectively, for all 58 lesions between OSEM (4 iteration, 8 subsets, 4mm Gaussian post-filer) and OSEM with the PSF-model included in the system matrix (3 iterations, 21 subsets, 2mm Gaussian) for image reconstruction. Armstrong et al. (19) reported that SUV_max_ and SUV_mean_ increased by 49% and 23%, respectively, in 68 lung cancer patients when PSF was used. Aklan et al. (20) reported that the mean difference in the relative change of SUV_max_ was 52±31% and the mean difference in SUV_mean_ was 24±17% between the non-PSF and PSF images of all lesions from 20 patients. The effect was more prominent for small lesions. However, none of these cited studies examined the effect of the applied reconstruction parameters.

Previous studies reported that PSF correction improved the PET image quality and lesion detection (9, 21). Thus, the PSF correction is considered to be useful for the qualitative interpretation of PET images. On the other hand, the present study suggested that PSF correction disturbed the quantitative accuracy of the PET images. This is the major drawback of PET images to be used as a biomarker. When the PSF correction is applied for PET image reconstruction, the mean value of SUV should be measured using ROIs with some extent. Harmonization using an appropriate smoothing filter may also be required for the images reconstructed by PSF algorithm.

The present study has some limitations. First, partial volume effects may affect the edge artifact because the images were reconstructed using a slice thickness of 5 mm. Second, a cone-shaped phantom was used while most tumors are irregularly shaped. The effect of the PSF algorithm may depend on the shape of the tumor. Finally, further clinical studies are necessary to evaluate the edge artifacts.

## Conclusion

PSF-based PET image reconstruction resulted in edge artifacts, the degree of which was related to pixel size, number of iterations, FWHM of the Gaussian filter, and object size.
